# The neurobiology of impulse control disorders in Parkinson’s disease: from neurotransmitters to neural networks

**DOI:** 10.1007/s00441-017-2771-0

**Published:** 2018-01-30

**Authors:** Chris Vriend

**Affiliations:** 1 Department of Psychiatry, VU University Medical Center, Amsterdam, The Netherlands; 2 Department of Anatomy & Neurosciences, VU University Medical Center, p/a sec. ANW O|2, PO Box 7007, 1007MB BT Amsterdam, the Netherlands; 3 Amsterdam Neuroscience, Amsterdam, The Netherlands

**Keywords:** Parkinson’s disease, Impulse control disorder, Neurobiology, Neuroimaging, Dopamine

## Abstract

Impulse control disorders (ICD) are common neuropsychiatric disorders that can arise in Parkinson’s disease (PD) patients after commencing dopamine replacement therapy. Approximately 15% of all patients develop these disorders and many more exhibit subclinical symptoms of impulsivity. ICD is thought to develop due to an interaction between the use of dopaminergic medication and an as yet unknown neurobiological vulnerability that either pre-existed before PD onset (possibly genetic) or is associated with neural alterations due to the PD pathology. This review discusses genes, neurotransmitters and neural networks that have been implicated in the pathophysiology of ICD in PD. Although dopamine and the related reward system have been the main focus of research, recently, studies have started to look beyond those systems to find new clues to the neurobiological underpinnings of ICD and come up with possible new targets for treatment. Studies on the whole-brain connectome to investigate the global alterations due to ICD development are currently lacking. In addition, there is a dire need for longitudinal studies that are able to disentangle the contributions of individual (genetic) traits and secondary effects of the PD pathology and chronic dopamine replacement therapy to the development of ICD in PD.

## Introduction

It is nowadays generally appreciated that Parkinson’s disease (PD) should not be regarded as merely a movement disorder. PD patients also commonly suffer from a broad range of non-motor symptoms. Of these, neuropsychiatric disorders are the most salient. Examples include anxiety, depression, apathy, psychosis and impulse control disorders (ICD) (Cooney and Stacy 2016). Studies in the last two decades have increasingly implicated the PD pathology, including degeneration of neurotransmitters (e.g., dopamine, serotonin or acetylcholine) in the pathophysiology of PD-related neuropsychiatric symptoms (Ceravolo et al. 2013; Frisina et al. 2009; Lim et al. 2009; Politis et al. 2010; Remy et al. 2005; Thobois et al. 2010; Voon et al. 2014; Vriend et al. 2014a, c). Strategies to manage these symptoms are therefore focused on optimising the dosage of dopaminergic medication and prescribing adjuvant pharmacotherapeutics (e.g., selective serotonin inhibitors or acetylcholinesterase inhibitors) (Connolly and Lang 2014; Seppi et al. 2011). Unlike apathy, depression and certain forms of anxiety, ICD does not improve with dopaminergic medication. In fact, development of ICD in PD has been consistently linked to the use of dopaminergic replacement therapy, most notably dopamine (D3) agonists (Joutsa et al. 2012a; Vela et al. 2016; Weintraub et al. 2010, 2013). Prevalence rates of ICD in unmedicated PD patients are similar to those of the general population (Weintraub et al. 2013). Approximately 15% of all medicated PD patients suffer from one or more clinically-significant ICD (Weintraub et al. 2010) but more patients screen positive for subclinical symptoms of ICD (Joutsa et al. 2012b; Vela et al. 2016; Vriend et al. 2014a). Examples of ICD include hypersexuality, pathological gambling, binge eating disorder and compulsive shopping (Weintraub et al. 2015). The prevalence rates of these ICDs seem to depend, among others, on cultural factors and gender (Maloney et al. 2017; Weintraub et al. 2015). Problematic internet use has also recently been suggested as a type of PD-related ICD (Wu et al. 2014). Phenomena related to ICD are hobbyism, punding, hoarding and dopamine dysregulation syndrome (DDS) but these are thought to have a distinct pathophysiology and risk profile (Weintraub et al. 2015). Besides the use of dopamine replacement therapy, other risk factors for ICD development include male gender, early disease onset, personal or family history of (behavioral) addiction, depression and point-mutations in certain dopamine or glutamate receptor genes (Bastiaens et al. 2013; Joutsa et al. 2012b; Lee et al. 2009; Weintraub et al. 2010). Management of ICD mainly consists of reducing the dosage of dopamine replacement therapy but this may give rise to dopamine withdrawal syndrome and exacerbate motor and psychiatric symptoms (Ramirez-Zamora et al. 2016). A systematic review concluded that there is currently insufficient evidence for an effective treatment (Tanwani et al. 2015; but see Ramirez-Zamora et al. 2016). For more details about the clinical aspects of ICD, see Weintraub et al. (2015).

Despite the relatively high prevalence rates of ICD in medicated PD patients, a large proportion do not develop ICD. This reflects the high heterogeneity of PD (van Balkom et al. 2016) and also indicates that ICD develops due to an interaction between dopamine replacement therapy and an as yet unknown neurobiological vulnerability. This review focuses on the neurobiological underpinnings of ICD and its development on multiple spatial scales, from genes and neurotransmitters, to brain regions and networks.

## Dopamine

Given the well-documented link between dopamine replacement therapy and ICD development in PD, it seems natural to suspect a direct involvement of (alterations in) the dopamine system. Before we delve into studies that implicate the dopamine system in the pathophysiology of ICD, it must be noted that dopaminergic medication—specifically dopamine D2-family receptor agonists—may also increase impulsivity by preventing pauses in D2-signaling through phasic endogenous dopamine release in the reward-related ventral striatum (Frank et al. 2004; Gerlach et al. 2003). In healthy conditions, these pauses in phasic dopamine release signal negative outcome after a particular behavioral response and tells the individual to avoid this behavior in the future (e.g. money loss after gambling) (van Eimeren et al. 2009). By preventing punishment learning but leaving positive reinforcement intact (which activates dopamine D1 receptors), dopaminergic medication impairs the encoding of harmful behavior and increases the probability of impulsive behavior and behavioral addiction. Studies in both medicated PD patients (e.g., Cools et al. 2006; Muhammed et al. 2016; Van Wouwe et al. 2017) and animal models (Cocker et al. 2017; Johnson et al. 2011; Rokosik and Napier 2012; Tremblay et al. 2017) have shown the impulsivity inducing effects of dopaminergic therapy. Nevertheless, the effects seems to depend on certain cllinical and neural characteristics (e.g., baseline dopamine levels) (Claassen et al. 2011; Cools and D’Esposito 2011) and on the type of task (Voon et al. 2017). For a more thorough review on the effects of dopamine replacement therapy on impulsive behavior in PD see Voon et al. (2017). Nevertheless, although these studies show that dopaminergic medication precipitates impulsive behavior in PD patients, their use does not seem sufficient to cause clinically significant ICD.

### Dopaminergic vulnerability to ICD development

PD patients with ICD (PD + ICD) exhibit several alterations in the dopamine system that may make them more vulnerable to the impulsivity inducing effects of dopamine replacement therapy. On the genetic level, multiple studies have associated gene polymorphisms of components of the dopamine signaling cascade with ICD but results have been mixed and generally based on small samples (Krishnamoorthy et al. 2016; Lee et al. 2009; Vallelunga et al. 2012; Zainal Abidin et al. 2015). Multiple cross-sectional molecular imaging studies with various radiotracers for the dopamine system have compared PD + ICD with PD patients without ICD (PD − ICD) and have provided some important insights into this system’s role in ICD. Nevertheless, some caution is advised when interpreting findings from molecular imaging studies. The resolution of molecular imaging scans is relatively coarse and makes interpretation about fine-grained structures (e.g., in the midbrain) difficult, e.g., due to spillover effects. Furthermore, binding of a radiotracer to a receptor always has to compete with the endogenous neurotransmitter and therefore between-group differences may be explained by alterations in the endogenous release of dopamine, altered availability of the receptor or a combination of the two. PD + ICD have been reported to exhibit higher endogenous dopamine release in the ventral striatum during performance of a gambling task (Steeves et al. 2009) or while viewing reward-related visual cues (O’Sullivan et al. 2011; Wu et al. 2015) compared with PD − ICD. PD + ICD also show lower ‘resting-state’ binding of [11C]Raclopride (a selective tracer for the D2 receptor) (Steeves et al. 2009) and lower binding of [11C]-(+)-PHNO (a D3 receptor selective tracer) in the ventral striatum (Payer et al. 2015). In addition, lower [11C] FLB-457 binding in the midbrain to D2/3 autoreceptors was observed during a gambling task (Ray et al. 2012). Lower D2/3 receptor availability is also found in studies on ICD or addiction in non-PD samples (Boileau et al. 2013; Volkow et al. 2002, 2008) and animal models (Dalley et al. 2007; Nader et al. 2006). Cross-sectional single photon emission computed tomography and positron emission tomography studies on the dopamine transporter (DAT) - that clears dopamine from the synaptic cleft after its release - have consistently shown reduced striatal DAT availability in PD + ICD compared with PD − ICD (Cilia et al. 2010; Lee et al. 2014; Voon et al. 2014). The problem with all studies that cross-sectionally compare PD + ICD with PD-ICD is that it is unclear if the neurobiological differences are due to already pre-existing neural characteristics (before PD or ICD onset), effects of prolonged dopaminergic therapy or alterations associated with ICD development. In that light, a retrospective follow-up study was performed that showed that lower DAT availability in the ventral striatum of medication naïve PD patients predated the development of ICD after commencing dopamine replacement therapy (Vriend et al. 2014a). Later, this finding was partly corroborated by a large prospective cohort study of PD patients that were medication-naïve at inclusion. A larger decrease in striatal DAT availability within the first year after inclusion increased the risk of ICD development after commencing dopamine replacement therapy (Smith et al. 2016). The reduced DAT availability may be interpreted as a (genetically) lower presence of DAT in the presynaptic dopamine neuron or more pronounced dopamine degeneration (Scherfler et al. 2007). In a previous review, the author postulated a tentative neurobiological model for the development of ICD, with a prominent role for degeneration of dopamine projections to the reward-related ventral striatum (Vriend et al. 2014b). This view, however, is at odds with the prevailing ‘overdose theory’ for ICD development, which states that the dosage of dopamine agonists used to supplement the loss of dopamine in the putamen to treat the motor symptoms overdoses the relatively preserved ventral striatum with dopamine (Voon et al. 2011b). As a result, postsynaptic dopamine D2-like receptors are overstimulated, leading to an imbalance between the ‘on’ and ‘off’ pathways of the reward-related cortico-striatal-thalamocortical circuit, thereby promoting impulsivity. For more information about the normal physiology of the cortico-striatal-thalamocortical circuits and the neuromodulatory role of dopamine see Gerfen and Surmeier (2011) and Haber and Knutson (2010). For more details on the overdose theory, see Voon et al. (2011b). The overdose theory is based on the observation that striatal dopamine denervates along a ‘caudo-rostral’ gradient with the posterior putamen being affected first and the ventral striatum only becoming affected in the later stages of the disease (Damier et al. 1999; Hsiao et al. 2014a; Kish et al. 1988). Due to the compensatory mechanisms of the brain, motor symptoms do not develop until approximately 50–60% of the nigrostriatal dopamine projections are degenerated (Cheng et al. 2010; Fearnley and Lees 1991).The threshold for the development of ICD is, however, unknown. It is therefore possible that the risk of ICD development is already increased at a lower percentage (e.g., 30%) of degeneration of dopaminergic projections to the ventral striatum. Whether or not ventral striatal dopamine degeneration plays a role in ICD development could be investigated with radiotracers selective for the presynaptic vesicular monoamine transporter 2 (VMAT2). VMAT2 is responsible for the uptake and storage of monoamines in vesicles prior to release at the presynaptic terminal (Bohnen et al. 2006) and may be less influenced by compensatory changes that can occur after dopamine degeneration compared with DAT (Bohnen et al. 2006; Hsiao et al. 2014b; Okamura et al. 2010). No study has yet investigated VMAT2 availability in relation to ICD in PD.

In summary, there is ample and still increasing evidence that heightened reward-related ventral striatal dopamine signaling, particularly through D3 receptors, is involved in the pathophysiology of ICD in PD. What is, however, less evident is whether or not this *hyperdopaminergic* state is due to pre-existing variants in components of the dopamine signaling cascade, associated with alterations due to the PD pathology or secondary effects of chronic dopaminergic treatment, or ICD development.

## Beyond dopamine

The ascending PD pathology also affects and eventually destroys, among others, serotonin- and noradrenalin-producing neurons (Braak et al. 2003). These neurotransmitter systems may also play a role in impulsive behavior and the pathophysiology of ICD. Most evidence for a role of serotonin in impulsive behavior comes from animal studies. It must be noted, however, that impulsivity is an umbrella term and encompasses multiple different subdivisions of impulsive behavior that are differently affected by serotonin signaling (Pattij and Schoffelmeer 2014; Winstanley et al. 2004). Enhancement of serotonin signaling, e.g., with selective serotonin reuptake inhibitors (SSRIs), reduces impulsive action in the five-choice serial reaction time task (Baarendse and Vanderschuren 2012; Homberg et al. 2007) but has no effect on impulsive choice in the delayed reward task (Baarendse and Vanderschuren 2012) and action cancelation in the stop-signal task (Bari et al. 2009). Similarly, lesioning serotonin-producing neurons in the raphe nucleus decreases inhibitory control in the five-choice serial reaction time task (Winstanley et al. 2004) and Go/No-Go task (Harrison et al. 1999) but has no effect on impulsive choice (Winstanley et al. 2004) or action cancelation (Eagle et al. 2009). In healthy humans, the SSRI citalopram enhanced activity of inhibition-related brain areas during a Go/No-Go task but had no effect on behavioral performance (Del-Ben et al. 2005; Macoveanu et al. 2012). Modulating serotonin levels during action cancelation also does not affect inhibitory control in humans (Chamberlain et al. 2006; Nandam et al. 2011), although these results may also partly reflect individual differences in the (integrity of the) serotonin system (Macoveanu et al. 2012; Ye et al. 2014). In PD - ICD patients, citalopram decreased action cancelation during the stop-signal task and enhanced task-related brain activation of the inferior frontal gyrus (Ye et al. 2014). Nevertheless, this effect was only observed in advanced stage PD patients. Only one single small study has investigated the role of serotonin in ICD in PD patients and found an association between polymorphisms in the 5-HT_2a_ receptor gene and ICD (Lee et al. 2009).

The noradrenergic system has not been studied in relation to ICD development in PD. In PD patients without psychiatric disorders, however, atomoxetine, a selective noradrenalin reuptake inhibitor (SNRI), was able to increase inhibition-related activity in the inferior frontal gyrus (Ye et al. 2015). In correspondence with a study in healthy controls (Nandam et al. 2011), atomoxetine had no overall effect on behavioral performance but was associated with behavioral improvement in those subjects that showed a more pronounced increase in inferior frontal gyrus activation (Ye et al. 2015). In a double-blind placebo-controlled study in PD patients, atomoxetine was able to improve action cancelation accuracy and reduce reflection impulsivity and risk taking (Kehagia et al. 2014). The link between the noradrenergic system and impulsive behavior has been studied more extensively in animal models. Overall, it can be stated that increases in noradrenergic signaling improve impulse control (Baarendse et al. 2013; Chamberlain et al. 2007; Robinson et al. 2008) but the mechanism is still unknown and is partly dependent on the subtype of impulsive behavior (see Bari and Robbins 2013 for a review).

Another neurotransmitter system that has recently been linked to ICD in PD is the opioid system. Opioid receptor antagonists, e.g., naltrexone, have been found to reduce symptoms in non-PD patients with alcohol addiction (Rosner et al. 2010), pathological gambling (Grant et al. 2008) and in other disorders within the impulsive-compulsive spectrum (see Piquet-Pessoa and Fontenelle, 2016 for a review). In PD + ICD patients, naltrexone had no effect on a clinician-rated global change score in ICD severity (primary outcome measure) but only a small and not clinically relevant effect on self-reported ICD symptom severity relative to placebo (Papay et al. 2014). The neurobiological mechanism of action of naltrexone is currently unknown but for alcohol addiction it has been suggested that it reduces reward-related mesolimbic dopamine release (Middaugh et al. 2003).

In conclusion, other neurotransmitters besides dopamine may be implicated in the pathophysiology of ICD in PD, although studies on this subject are scarce. Furthermore, there are complex interactions between the dopamine, serotonin and noradrenalin system (e.g., dopamine and noradrenalin rely on the same biosynthethic pathway) (Benarroch 2009; Boureau and Dayan 2010; Rommelfanger and Weinshenker 2007) and (pharmacologically) influencing one system will also have consequences for another. This hampers clear-cut interpretation of study results.

Nevertheless, given the results from animal studies and preliminary evidence that suggest that serotonergic, noradrenergic or opioid drugs can ameliorate inhibitory control in PD, more studies into these systems are warranted.

## Imaging studies on brain structure and function during task and rest

Although few, a number of studies have used functional neuroimaging techniques to examine brain alterations associated with ICD at the local and network level. Given the nature of ICD, neural activation studies have mainly focused on reward-based tasks. Presentation of reward-related visual cues during fMRI increased activation of the ventral striatum of PD patients with pathological gambling (Frosini et al. 2010) or hypersexuality (Politis et al. 2013) compared with those without. Conversely, during risk taking neural activity in the ventral striatum is decreased in PD + ICD compared with PD - ICD (Rao et al. 2010; Voon et al. 2011a). On the other hand, the only resting state fMRI study published to date showed no alterations between PD + ICD and PD − ICD in the connectivity of the ventral striatum with other reward-related brain areas (Carriere et al. 2015). They did, however, observe between-group differences in the connectivity of the associative striatum (anterior putamen, dorsal caudate) and to a lesser extent the motor striatum (posterior putamen). The anterior putamen showed less connectivity with, among others, the inferior temporal and frontal gyrus and anterior cingulate gyrus, while the dorsal caudate showed less connectivity with the orbitofrontal cortex, middle frontal and inferior temporal gyrus. The lower connectivity of the associative striatum in PD + ICD patients versus PD − ICD patients is consistent with the theoretical framework from Mary Phillips and colleagues on the neural basis of affective disorders, stating that an imbalance between an underactive dorsal—associative−control system and overactive ventral—limbic—emotion system can give rise to neuropsychiatric symptoms (Phillips et al. 2003, 2008). The associative striatum and connected areas are also highly involved in cognitive functions, especially executive functions (Monchi et al. 2007; O’Callaghan et al. 2014) and the reduced connectivity of the associative striatum might therefore also underlie some of the reports on executive dysfunction in PD + ICD patients (Santangelo et al. 2009; Vitale et al. 2011). Nevertheless, studies on the cognitive functions of PD + ICD compared with PD − ICD have also provided null findings (Antonini et al. 2017; Djamshidian et al. 2011).

Diffusion Tensor Imaging (DTI) provides a measure for the integrity of white matter tracts. An often used diffusion parameter as a marker for white matter integrity is fractional anisotropy (FA), which represents diffusion along the axon relative to two orthogonal radial directions (Alexander et al. 2007; Rae et al. 2012). Compared with healthy controls, PD − ICD had lower FA in white matter bundles in orbitofrontal, anterior cingulate and medial prefrontal brain areas. PD + ICD patients showed no such between-group difference compared with healthy controls. Compared with PD − ICD patients, PD + ICD patients showed higher FA in a number of fiber tracts, including the anterior corpus callosum, posterior limb of the internal capsule and thalamic radiation and no areas with decreased FA. The authors speculate that overuse of reward-related prefrontal brain areas results in relative preservation or even greater directivity of the white matter tracts in PD + ICD. They do note, however, that their findings must be regarded as preliminary given the small sample size.

Structural alterations have also been studied using cortical thickness or voxel-based morphometry analyses. In the largest study to date (58 PD + ICD vs 52 PD − ICD), cortical thinning was observed in PD + ICD versus PD − ICD patients in the superior orbitofrontal gyrus, rostral middle frontal gyrus and bilateral caudal middle frontal region, concomitant with lower volume of the accumbens but increased volume of the amygdala (Biundo et al. 2015). Additionally, volumes of the rostral middle frontal, the inferior parietal and supramarginal area were positively associated with the severity of ICD symptoms. It must be noted, however, that PD + ICD and PD − ICD patients also differed in age and disease duration, which may have confounded these findings. Several smaller studies have also been conducted (Biundo et al. 2011; Carriere et al. 2015; Cerasa et al. 2014; Pellicano et al. 2015; Tessitore et al. 2016). Those studies that observed between-group differences (either increased or decreased volume/thickness) have generally observed those in reward-related brain areas. It is however unclear if these findings should be interpreted as a pre-existing trait, maladaptive structural changes in response to chronic dopaminergic stimulation (Pellicano et al. 2015) or neuroplastic changes in the reward system due to frequent exposure to appetitive stimuli. It is also unknown if and how these structural alterations contribute to deficits in inhibitory control (Tessitore et al. 2016).

In conclusion, various functional and structural imaging studies have investigated ICD in PD. These studies generally implicate the reward-related prefrontal and striatal areas, although there are inconsistencies in the direction of the effect and null findings have also been reported. This is partly due to the small sample sizes of individual studies.

## Conclusions and future prospects

**Fig. 1:**
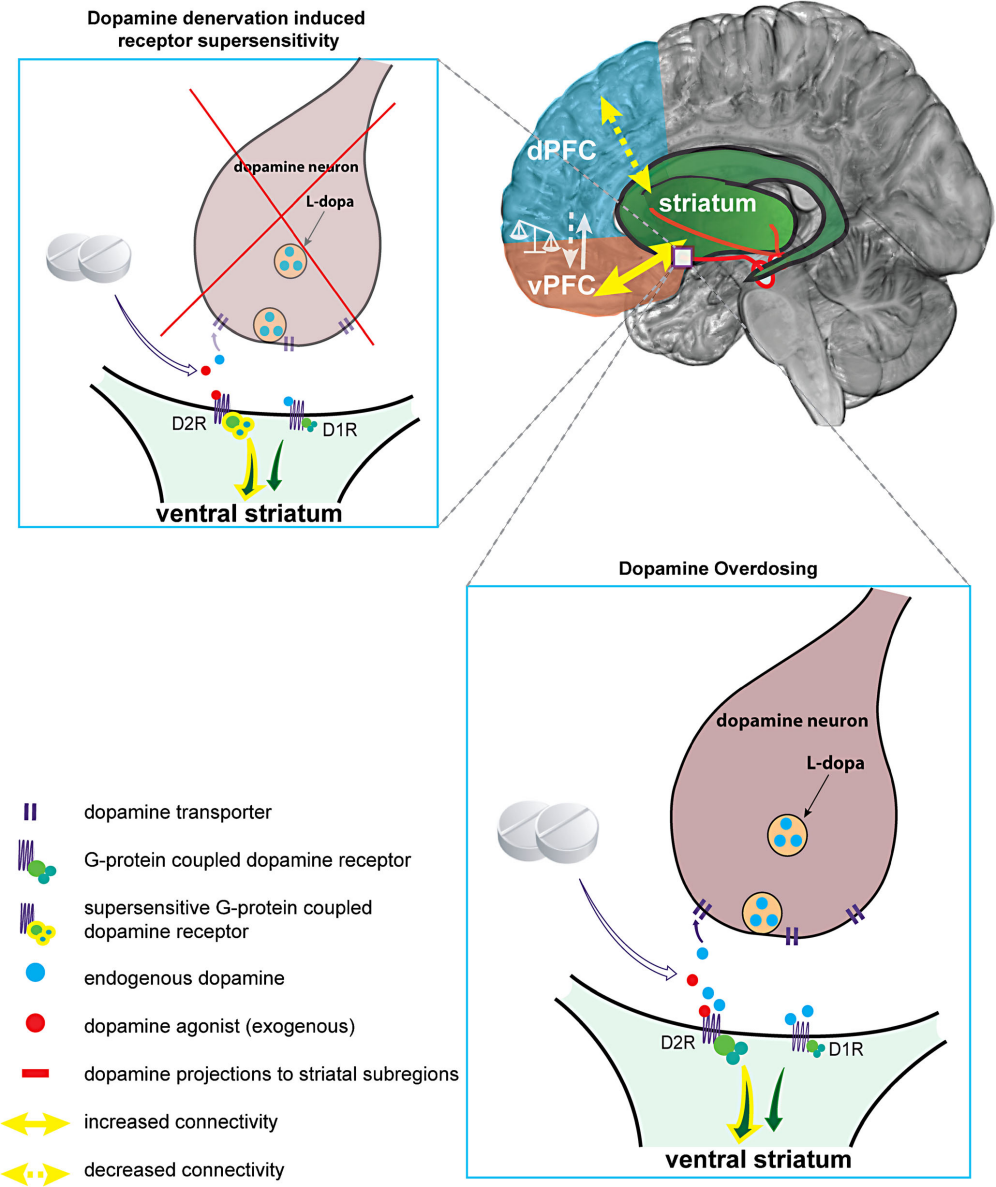
Illustrative summary of putative neurobiological mechanisms for ICD development. Two proposed neurobiological mechanisms concerning the dopamine system and dopamine suppletion are illustrated: (1) Dopamine denervation-induced receptor supersensitivity (*upper left*) and (2) dopamine overdosing (*lower right*). (1) When dopamine projections from the midbrain towards the ventral striatum degenerate in PD, postsynaptic D2-like dopamine receptors (especially D3 receptors), can develop a heightened sensitivity for endogenous and exogenous dopamine (Prieto et al. 2011). Treatment with dopaminergic medication for PD may subsequently lead to an exaggerated response in post-synaptic reward-related ventral striatal neurons and increased activity in connected limbic areas, including the ventral prefrontal cortex (*vPFC*); see Vriend et al. (2014b) for more details. (2) The overdose theory assumes that dopamine projections towards the ventral striatum are still relatively spared compared with projections towards the dorsal ‘motor’ striatum for which dopaminergic medication is titrated. As a result, dopaminergic medication overdoses post-synaptic D2-like receptors in the ventral striatum, leading to increased activity in the connected reward-related brain areas; see Cools (2006) and Voon et al. (2011b) for more details. *Upper right* Apart from aberrations in reward-related areas, reduced connectivity of the dorsal associative striatum and dorsal PFC (Carriere et al. 2015) may impede proper regulation of impulsive behavior and further promote ICD development. This imbalance between dorsal and ventral PFC is consistent with the framework of Phillips et al. (2003, 2008) on the development of neuropsychiatric disorders in non-PD samples

Based on an abundance of research, it seems clear that ICD in PD is linked to a *hyperdopaminergic* state. Whether this is due to supplementing dopamine to a relatively spared ventral striatum, i.e., ‘overdose theory’ (Cools 2006; Voon et al. 2011b) or dopamine denervation-induced receptor supersensitivity of D3 receptors in the ventral striatum (Prieto et al. 2011; Vriend et al. 2014b) is still unknown (Fig. 1). It has also been argued that the PD pathology is not or only a minor contributor to the development of ICD because there are also reported cases of ICD in non-PD patients treated with dopaminergic drugs (e.g., restless legs syndrome) (Tippmann-Peikert et al. 2007). Although it is true that PD patients without medication are not at increased risk of developing ICD compared with the general population (Weintraub et al. 2013), unmedicated PD-ICD patients do exhibit subclinical inhibitory deficits (Obeso et al. 2011; Vriend et al. 2015) or altered reward sensitivity (Aarts et al. 2012) and medicated PD + ICD and matched PD − ICD are equally impaired in learning from punishment compared with healthy controls (Leplow et al. 2017). This suggests that the effects of the PD pathology on the dopamine system do play a role in ICD development. ICD subtypes and different aspects of impulse control might be differentially affected by dopamine degeneration and dopamine suppletion (see Voon et al. 2017 for a review).

It is also important to consider the role of the serotonergic and noradrenergic systems since these are also affected by the PD pathology and implicated in impulse control. Dopaminergic approaches to treat ICD often fail because adjusting the dopaminergic medication dosage can prompt withdrawal symptoms or exacerbate motor and psychiatric symptoms (Ramirez-Zamora et al. 2016). New treatments for ICD by modulating the serotonergic and noradrenergic, or possibly the opioid system, may circumvent this.

Lastly, neuroimaging can be a powerful tool to investigate neurobiological mechanisms and risk factors for ICD. Although the relatively few studies that have investigated structure and function of brain areas and networks involved in ICD have provided important insights, they are somewhat hampered by small sample sizes and heterogeneity in study designs, making cross-study comparisons (in a meta-analysis) challenging. Furthermore, except for two (Smith et al. 2016; Vriend et al. 2014a), all the reported studies were performed cross-sectionally, making it difficult to discern whether results are explained by pre-existing (genetic) traits or associated with pathological alterations due to the PD pathology, alterations due to chronic dopamine replacement therapy or their interaction. Answering this question warrants longitudinal neuroimaging studies that follow patients from the early unmedicated stages (or preferably even before PD onset). Because the development of ICD after commencing dopaminergic therapy is highly variable (Bastiaens et al. 2013), this would require following a large group of patients for multiple years.

## References

[CR1] Aarts E, Helmich RC, Janssen MJ, Oyen WJ, Bloem BR, Cools R (2012). Aberrant reward processing in Parkinson’s disease is associated with dopamine cell loss. NeuroImage.

[CR2] Alexander AL, Lee JE, Lazar M, Field AS (2007). Diffusion tensor imaging of the brain. Neurother: J Am Soc Exp Neurother Ther.

[CR3] Antonini A, Barone P, Bonuccelli U, Annoni K, Asgharnejad M, Stanzione P (2017). ICARUS study: prevalence and clinical features of impulse control disorders in Parkinson’s disease. J Neurol Neurosurg Psychiatry.

[CR4] Baarendse PJ, Vanderschuren LJ (2012). Dissociable effects of monoamine reuptake inhibitors on distinct forms of impulsive behavior in rats. Psychopharmacology.

[CR5] Baarendse PJ, Winstanley CA, Vanderschuren LJ (2013). Simultaneous blockade of dopamine and noradrenaline reuptake promotes disadvantageous decision making in a rat gambling task. Psychopharmacology.

[CR6] Bari A, Robbins TW (2013). Inhibition and impulsivity: behavioral and neural basis of response control. Prog Neurobiol.

[CR7] Bari A, Eagle DM, Mar AC, Robinson ES, Robbins TW (2009). Dissociable effects of noradrenaline, dopamine, and serotonin uptake blockade on stop task performance in rats. Psychopharmacology.

[CR8] Bastiaens J, Dorfman BJ, Christos PJ, Nirenberg MJ (2013). Prospective cohort study of impulse control disorders in Parkinson’s disease. Move Disord.

[CR9] Benarroch EE (2009). The locus ceruleus norepinephrine system: functional organization and potential clinical significance. Neurology.

[CR10] Biundo R, Formento-Dojot P, Facchini S, Vallelunga A, Ghezzo L, Foscolo L, Meneghello F, Antonini A (2011). Brain volume changes in Parkinson’s disease and their relationship with cognitive and behavioural abnormalities. J Neurol Sci.

[CR11] Biundo R, Weis L, Facchini S, Formento-Dojot P, Vallelunga A, Pilleri M, Weintraub D, Antonini A (2015). Patterns of cortical thickness associated with impulse control disorders in Parkinson’s disease. Move Disord.

[CR12] Bohnen NI, Albin RL, Koeppe RA, Wernette KA, Kilbourn MR, Minoshima S, Frey KA (2006). Positron emission tomography of monoaminergic vesicular binding in aging and Parkinson disease. J Cereb Blood Flow Metab.

[CR13] Boileau I, Payer D, Chugani B, Lobo D, Behzadi A, Rusjan PM, Houle S, Wilson AA, Warsh J, Kish SJ, Zack M (2013). The D2/3 dopamine receptor in pathological gambling: a positron emission tomography study with [11C]-(+)-propyl-hexahydro-naphtho-oxazin and [11C]raclopride. Addiction.

[CR14] Boureau Y-L, Dayan P (2010). Opponency revisited: competition and cooperation between dopamine and serotonin. Neuropsychopharmacology.

[CR15] Braak H, Del Tredici K, Rub U, de Vos RA, Jansen Steur EN, Braak E (2003). Staging of brain pathology related to sporadic Parkinson’s disease. Neurobiol Aging.

[CR16] Carriere N, Lopes R, Defebvre L, Delmaire C, Dujardin K (2015). Impaired corticostriatal connectivity in impulse control disorders in Parkinson disease. Neurology.

[CR17] Cerasa A, Salsone M, Nigro S, Chiriaco C, Donzuso G, Bosco D, Vasta R, Quattrone A (2014). Cortical volume and folding abnormalities in Parkinson’s disease patients with pathological gambling. Parkinsonism Relat Disord.

[CR18] Ceravolo R, Frosini D, Poletti M, Kiferle L, Pagni C, Mazzucchi S, Volterrani D, Bonuccelli U (2013). Mild affective symptoms in de novo Parkinson’s disease patients: relationship with dopaminergic dysfunction. Eur J Neurol.

[CR19] Chamberlain SR, Muller U, Blackwell AD, Clark L, Robbins TW, Sahakian BJ (2006). Neurochemical modulation of response inhibition and probabilistic learning in humans. Science.

[CR20] Chamberlain SR, Del Campo N, Dowson J, Muller U, Clark L, Robbins TW, Sahakian BJ (2007). Atomoxetine improved response inhibition in adults with attention deficit/hyperactivity disorder. Biol Psychiatry.

[CR21] Cheng HC, Ulane CM, Burke RE (2010). Clinical progression in Parkinson disease and the neurobiology of axons. Ann Neurol.

[CR22] Cilia R, Ko JH, Cho SS, van Eimeren T, Marotta G, Pellecchia G, Pezzoli G, Antonini A, Strafella AP (2010). Reduced dopamine transporter density in the ventral striatum of patients with Parkinson’s disease and pathological gambling. Neurobiol Dis.

[CR23] Claassen DO, van den Wildenberg WP, Ridderinkhof KR, Jessup CK, Harrison MB, Wooten GF, Wylie SA (2011). The risky business of dopamine agonists in Parkinson disease and impulse control disorders. Behav Neurosci.

[CR24] Cocker PJ, Tremblay M, Kaur S, Winstanley CA (2017). Chronic administration of the dopamine D2/3 agonist ropinirole invigorates performance of a rodent slot machine task, potentially indicative of less distractible or compulsive-like gambling behaviour. Psychopharmacology.

[CR25] Connolly BS, Lang AE (2014). Pharmacological treatment of Parkinson disease: a review. JAMA.

[CR26] Cools R (2006). Dopaminergic modulation of cognitive function-implications for L-DOPA treatment in Parkinson’s disease. Neurosci Biobehav Rev.

[CR27] Cools R, D’Esposito M (2011). Inverted-U-shaped dopamine actions on human working memory and cognitive control. Biol Psychiatry.

[CR28] Cools R, Altamirano L, D’Esposito M (2006). Reversal learning in Parkinson’s disease depends on medication status and outcome valence. Neuropsychologia.

[CR29] Cooney JW, Stacy M (2016). Neuropsychiatric issues in Parkinson’s disease. Curr Neurol Neurosci Rep.

[CR30] Dalley JW, Fryer TD, Brichard L, Robinson ES, Theobald DE, Laane K, Pena Y, Murphy ER, Shah Y, Probst K, Abakumova I, Aigbirhio FI, Richards HK, Hong Y, Baron JC, Everitt BJ, Robbins TW (2007). Nucleus accumbens D2/3 receptors predict trait impulsivity and cocaine reinforcement. Science.

[CR31] Damier P, Hirsch EC, Agid Y, Graybiel AM (1999). The substantia nigra of the human brain. II. Patterns of loss of dopamine-containing neurons in Parkinson’s disease. Brain.

[CR32] Del-Ben CM, Deakin JF, McKie S, Delvai NA, Williams SR, Elliott R, Dolan M, Anderson IM (2005). The effect of citalopram pretreatment on neuronal responses to neuropsychological tasks in normal volunteers: an FMRI study. Neuropsychopharmacology.

[CR33] Djamshidian A, O’Sullivan SS, Lees A, Averbeck BB (2011). Stroop test performance in impulsive and non impulsive patients with Parkinson’s disease. Parkinsonism Relat Disord.

[CR34] Eagle DM, Lehmann O, Theobald DE, Pena Y, Zakaria R, Ghosh R, Dalley JW, Robbins TW (2009). Serotonin depletion impairs waiting but not stop-signal reaction time in rats: implications for theories of the role of 5-HT in behavioral inhibition. Neuropsychopharmacology.

[CR35] Fearnley JM, Lees AJ (1991). Ageing and Parkinson’s disease: substantia nigra regional selectivity. Brain.

[CR36] Frank MJ, Seeberger LC, O’Reilly RC (2004). By carrot or by stick: cognitive reinforcement learning in parkinsonism. Science.

[CR37] Frisina PG, Haroutunian V, Libow LS (2009). The neuropathological basis for depression in Parkinson’s disease. Parkinsonism Relat Disord.

[CR38] Frosini D, Pesaresi I, Cosottini M, Belmonte G, Rossi C, Dell’Osso L, Murri L, Bonuccelli U, Ceravolo R (2010) Parkinson’s disease and pathological gambling: results from a functional MRI study. Move Disord 25:2449–2453 10.1002/mds.23369 20976739

[CR39] Gerfen CR, Surmeier DJ (2011). Modulation of striatal projection systems by dopamine. Annu Rev Neurosci.

[CR40] Gerlach M, Double K, Arzberger T, Leblhuber F, Tatschner T, Riederer P (2003). Dopamine receptor agonists in current clinical use: comparative dopamine receptor binding profiles defined in the human striatum. J Neural Transm.

[CR41] Grant JE, Kim SW, Hartman BK (2008). A double-blind, placebo-controlled study of the opiate antagonist naltrexone in the treatment of pathological gambling urges. J Clin Psychiatry.

[CR42] Haber SN, Knutson B (2010). The reward circuit: linking primate anatomy and human imaging. Neuropsychopharmacology.

[CR43] Harrison AA, Everitt BJ, Robbins TW (1999). Central serotonin depletion impairs both the acquisition and performance of a symmetrically reinforced go/no-go conditional visual discrimination. Behav Brain Res.

[CR44] Homberg JR, Pattij T, Janssen MC, Ronken E, De Boer SF, Schoffelmeer AN, Cuppen E (2007). Serotonin transporter deficiency in rats improves inhibitory control but not behavioural flexibility. Eur J Neurosci.

[CR45] Hsiao IT, Weng YH, Hsieh CJ, Lin WY, Wey SP, Kung MP, Yen TC, Lu CS, Lin KJ (2014). Correlation of Parkinson disease severity and 18F-DTBZ positron emission tomography. JAMA Neurol.

[CR46] Hsiao IT, Weng YH, Lin WY, Hsieh CJ, Wey SP, Yen TC, Kung MP, Lu CS, Lin KJ (2014). Comparison of 99mTc-TRODAT-1 SPECT and 18 F-AV-133 PET imaging in healthy controls and Parkinson’s disease patients. Nucl Med Biol.

[CR47] Johnson PS, Madden GJ, Brewer AT, Pinkston JW, Fowler SC (2011). Effects of acute pramipexole on preference for gambling-like schedules of reinforcement in rats. Psychopharmacology.

[CR48] Joutsa J, Martikainen K, Vahlberg T, Kaasinen V (2012). Effects of dopamine agonist dose and gender on the prognosis of impulse control disorders in Parkinson’s disease. Parkinsonism Relat Disord.

[CR49] Joutsa J, Martikainen K, Vahlberg T, Voon V, Kaasinen V (2012). Impulse control disorders and depression in Finnish patients with Parkinson’s disease. Parkinsonism Relat Disord.

[CR50] Kehagia AA, Housden CR, Regenthal R, Barker RA, Muller U, Rowe J, Sahakian BJ, Robbins TW (2014). Targeting impulsivity in Parkinson’s disease using atomoxetine. Brain.

[CR51] Kish SJ, Shannak K, Hornykiewicz O (1988). Uneven pattern of dopamine loss in the striatum of patients with idiopathic Parkinson’s disease. Pathophysiologic and clinical implications. N Engl J Med.

[CR52] Krishnamoorthy S, Rajan R, Banerjee M, Kumar H, Sarma G, Krishnan S, Sarma S, Kishore A (2016). Dopamine D3 receptor Ser9Gly variant is associated with impulse control disorders in Parkinson’s disease patients. Parkinsonism Relat Disord.

[CR53] Lee JY, Lee EK, Park SS, Lim JY, Kim HJ, Kim JS, Jeon BS (2009). Association of DRD3 and GRIN2B with impulse control and related behaviors in Parkinson’s disease. Move Disord.

[CR54] Lee JY, Seo SH, Kim YK, Yoo HB, Kim YE, Song IC, Lee JS, Jeon BS (2014). Extrastriatal dopaminergic changes in Parkinson’s disease patients with impulse control disorders. J Neurol Neurosurg Psychiatry.

[CR55] Leplow B, Sepke M, Schonfeld R, Pohl J, Oelsner H, Latzko L, Ebersbach G (2017). Impaired learning of punishments in Parkinson’s disease with and without impulse control disorder. J Neural Transm (Vienna).

[CR56] Lim SY, Fox SH, Lang AE (2009). Overview of the extranigral aspects of Parkinson disease. Arch Neurol.

[CR57] Macoveanu J, Hornboll B, Elliott R, Erritzoe D, Paulson OB, Siebner H, Knudsen GM, Rowe JB (2012). Serotonin 2A receptors, citalopram and tryptophan-depletion: a multimodal imaging study of their interactions during response inhibition. Neuropsychopharmacology.

[CR58] Maloney EM, Djamshidian A, O’Sullivan SS (2017). Phenomenology and epidemiology of impulsive-compulsive behaviours in Parkinson’s disease, atypical parkinsonian disorders and non-parkinsonian populations. J Neurol Sci.

[CR59] Middaugh LD, Szumlinski KK, Van Patten Y, Marlowe AL, Kalivas PW (2003). Chronic ethanol consumption by C57BL/6 mice promotes tolerance to its interoceptive cues and increases extracellular dopamine, an effect blocked by naltrexone. Alcohol Clin Exp Res.

[CR60] Monchi O, Petrides M, Mejia-Constain B, Strafella AP (2007). Cortical activity in Parkinson’s disease during executive processing depends on striatal involvement. Brain J Neurol.

[CR61] Muhammed K, Manohar S, Ben Yehuda M, Chong TT, Tofaris G, Lennox G, Bogdanovic M, Hu M, Husain M (2016). Reward sensitivity deficits modulated by dopamine are associated with apathy in Parkinson’s disease. Brain J Neurol.

[CR62] Nader MA, Morgan D, Gage HD, Nader SH, Calhoun TL, Buchheimer N, Ehrenkaufer R, Mach RH (2006). PET imaging of dopamine D2 receptors during chronic cocaine self-administration in monkeys. Nat Neurosci.

[CR63] Nandam LS, Hester R, Wagner J, Cummins TD, Garner K, Dean AJ, Kim BN, Nathan PJ, Mattingley JB, Bellgrove MA (2011). Methylphenidate but not atomoxetine or citalopram modulates inhibitory control and response time variability. Biol Psychiatry.

[CR64] Obeso I, Wilkinson L, Jahanshahi M (2011). Levodopa medication does not influence motor inhibition or conflict resolution in a conditional stop-signal task in Parkinson’s disease. Exp Brain Res.

[CR65] O’Callaghan C, Bertoux M, Hornberger M (2014). Beyond and below the cortex: the contribution of striatal dysfunction to cognition and behaviour in neurodegeneration. J Neurol Neurosurg Psychiatry.

[CR66] Okamura N, Villemagne VL, Drago J, Pejoska S, Dhamija RK, Mulligan RS, Ellis JR, Ackermann U, O’Keefe G, Jones G, Kung HF, Pontecorvo MJ, Skovronsky D, Rowe CC (2010). In vivo measurement of vesicular monoamine transporter type 2 density in Parkinson disease with (18)F-AV-133. J Nucl Med.

[CR67] O’Sullivan SS, Wu K, Politis M, Lawrence AD, Evans AH, Bose SK, Djamshidian A, Lees AJ, Piccini P (2011). Cue-induced striatal dopamine release in Parkinson’s disease-associated impulsive-compulsive behaviours. Brain J Neurol.

[CR68] Papay K, Xie SX, Stern M, Hurtig H, Siderowf A, Duda JE, Minger J, Weintraub D (2014). Naltrexone for impulse control disorders in Parkinson disease: a placebo-controlled study. Neurology.

[CR69] Pattij T, Schoffelmeer AN (2014) Serotonin and inhibitory response control: Focusing on the role of 5-HT receptors. Eur J Pharmacol 753:140–145 10.1016/j.ejphar.2014.05.064 25094037

[CR70] Payer DE, Guttman M, Kish SJ, Tong J, Strafella A, Zack M, Adams JR, Rusjan P, Houle S, Furukawa Y, Wilson AA, Boileau I (2015). [(1)(1)C]-(+)-PHNO PET imaging of dopamine D(2/3) receptors in Parkinson’s disease with impulse control disorders. Move Disord.

[CR71] Pellicano C, Niccolini F, Wu K, O’Sullivan SS, Lawrence AD, Lees AJ, Piccini P, Politis M (2015). Morphometric changes in the reward system of Parkinson’s disease patients with impulse control disorders. J Neurol.

[CR72] Phillips ML, Drevets WC, Rauch SL, Lane R (2003). Neurobiology of emotion perception II: implications for major psychiatric disorders. Biol Psychiatry.

[CR73] Phillips ML, Ladouceur CD, Drevets WC (2008). A neural model of voluntary and automatic emotion regulation: implications for understanding the pathophysiology and neurodevelopment of bipolar disorder. Mol Psychiatry.

[CR74] Piquet-Pessoa M, Fontenelle LF (2016). Opioid antagonists in broadly defined behavioral addictions: a narrative review. Expert Opin Pharmacother.

[CR75] Politis M, Wu K, Loane C, Turkheimer FE, Molloy S, Brooks DJ, Piccini P (2010). Depressive symptoms in PD correlate with higher 5-HTT binding in raphe and limbic structures. Neurology.

[CR76] Politis M, Loane C, Wu K, O’Sullivan SS, Woodhead Z, Kiferle L, Lawrence AD, Lees AJ, Piccini P (2013) Neural response to visual sexual cues in dopamine treatment-linked hypersexuality in Parkinson’s disease. Brain 136:400–411 10.1093/brain/aws326 23378222

[CR77] Prieto GA, Perez-Burgos A, Palomero-Rivero M, Galarraga E, Drucker-Colin R, Bargas J (2011). Upregulation of D2-class signaling in dopamine-denervated striatum is in part mediated by D3 receptors acting on ca V 2.1 channels via PIP2 depletion. J Neurophysiol.

[CR78] Rae CL, Correia MM, Altena E, Hughes LE, Barker RA, Rowe JB (2012). White matter pathology in Parkinson’s disease: the effect of imaging protocol differences and relevance to executive function. NeuroImage.

[CR79] Ramirez-Zamora A, Gee L, Boyd J, Biller J (2016). Treatment of impulse control disorders in Parkinson’s disease: practical considerations and future directions. Expert Rev Neurother.

[CR80] Rao H, Mamikonyan E, Detre JA, Siderowf AD, Stern MB, Potenza MN, Weintraub D (2010). Decreased ventral striatal activity with impulse control disorders in Parkinson’s disease. Mov Disord.

[CR81] Ray NJ, Miyasaki JM, Zurowski M, Ko JH, Cho SS, Pellecchia G, Antonelli F, Houle S, Lang AE, Strafella AP (2012). Extrastriatal dopaminergic abnormalities of DA homeostasis in Parkinson’s patients with medication-induced pathological gambling: a [11C] FLB-457 and PET study. Neurobiol Dis.

[CR82] Remy P, Doder M, Lees A, Turjanski N, Brooks D (2005). Depression in Parkinson’s disease: loss of dopamine and noradrenaline innervation in the limbic system. Brain J Neurol.

[CR83] Robinson ES, Eagle DM, Mar AC, Bari A, Banerjee G, Jiang X, Dalley JW, Robbins TW (2008). Similar effects of the selective noradrenaline reuptake inhibitor atomoxetine on three distinct forms of impulsivity in the rat. Neuropsychopharmacology.

[CR84] Rokosik SL, Napier TC (2012). Pramipexole-induced increased probabilistic discounting: comparison between a rodent model of Parkinson’s disease and controls. Neuropsychopharmacology.

[CR85] Rommelfanger KS, Weinshenker D (2007). Norepinephrine: the redheaded stepchild of Parkinson’s disease. Biochem Pharmacol.

[CR86] Rosner S, Hackl-Herrwerth A, Leucht S, Vecchi S, Srisurapanont M, Soyka M (2010) Opioid antagonists for alcohol dependence. Cochrane Database Syst Rev CD001867 10.1002/14651858.CD001867.pub3 PMC13590218 21154349

[CR87] Santangelo G, Vitale C, Trojano L, Verde F, Grossi D, Barone P (2009). Cognitive dysfunctions and pathological gambling in patients with Parkinson’s disease. Move Disord.

[CR88] Scherfler C, Schwarz J, Antonini A, Grosset D, Valldeoriola F, Marek K, Oertel W, Tolosa E, Lees AJ, Poewe W (2007). Role of DAT-SPECT in the diagnostic work up of parkinsonism. Move Disord.

[CR89] Seppi K, Weintraub D, Coelho M, Perez-Lloret S, Fox SH, Katzenschlager R, Hametner EM, Poewe W, Rascol O, Goetz CG, Sampaio C (2011). The Movement Disorder Society evidence-based medicine review update: treatments for the non-motor symptoms of Parkinson’s disease. Move Disord.

[CR90] Smith KM, Xie SX, Weintraub D (2016). Incident impulse control disorder symptoms and dopamine transporter imaging in Parkinson disease. J Neurol Neurosurg Psychiatry.

[CR91] Steeves TD, Miyasaki J, Zurowski M, Lang AE, Pellecchia G, Van Eimeren T, Rusjan P, Houle S, Strafella AP (2009). Increased striatal dopamine release in parkinsonian patients with pathological gambling: a [11C] raclopride PET study. Brain J Neurol.

[CR92] Tanwani P, Fernie BA, Nikcevic AV, Spada MM (2015). A systematic review of treatments for impulse control disorders and related behaviours in Parkinson’s disease. Psychiatry research 225:402-406Tessitore a, Santangelo G, de Micco R, Vitale C, Giordano a, Raimo S, Corbo D, Amboni M, Barone P, Tedeschi G (2016) cortical thickness changes in patients with Parkinson’s disease and impulse control disorders. Parkinsonism Relat Disord.

[CR93] Tessitore A, Santangelo G, De Micco R, Vitale C, Giordano A, Raimo S, Corbo D, Amboni M, Barone P, Tedeschi G (2016). Cortical thickness changes in patients with Parkinson’s disease and impulse control disorders. Parkinsonism Relat Disord.

[CR94] Thobois S, Ardouin C, Lhommee E, Klinger H, Lagrange C, Xie J, Fraix V, Coelho Braga MC, Hassani R, Kistner A, Juphard A, Seigneuret E, Chabardes S, Mertens P, Polo G, Reilhac A, Costes N, LeBars D, Savasta M, Tremblay L, Quesada JL, Bosson JL, Benabid AL, Broussolle E, Pollak P, Krack P (2010). Non-motor dopamine withdrawal syndrome after surgery for Parkinson’s disease: predictors and underlying mesolimbic denervation. Brain.

[CR95] Tippmann-Peikert M, Park JG, Boeve BF, Shepard JW, Silber MH (2007). Pathologic gambling in patients with restless legs syndrome treated with dopaminergic agonists. Neurology.

[CR96] Tremblay M, Silveira MM, Kaur S, Hosking JG, Adams WK, Baunez C, Winstanley CA (2017). Chronic D2/3 agonist ropinirole treatment increases preference for uncertainty in rats regardless of baseline choice patterns. Eur J Neurosci.

[CR97] Vallelunga A, Flaibani R, Formento-Dojot P, Biundo R, Facchini S, Antonini A (2012). Role of genetic polymorphisms of the dopaminergic system in Parkinson’s disease patients with impulse control disorders. Parkinsonism Relat Disord.

[CR98] van Balkom TD, Vriend C, Berendse HW, Foncke EM, van der Werf YD, van den Heuvel OA, Klein M (2016). Profiling cognitive and neuropsychiatric heterogeneity in Parkinson’s disease. Parkinsonism Relat Disord.

[CR99] van Eimeren T, Ballanger B, Pellecchia G, Miyasaki JM, Lang AE, Strafella AP (2009). Dopamine agonists diminish value sensitivity of the orbitofrontal cortex: a trigger for pathological gambling in Parkinson’s disease?. Neuropsychopharmacology.

[CR100] Van Wouwe NC, Claassen DO, Neimat JS, Kanoff KE, Wylie SA (2017). Dopamine selectively modulates the outcome of learning unnatural action-valence associations. J Cogn Neurosci.

[CR101] Vela L, Martinez Castrillo JC, Garcia Ruiz P, Gasca-Salas C, Macias Macias Y, Perez Fernandez E, Ybot I, Lopez Valdes E, Kurtis MM, Posada Rodriguez IJ, Mata M, Ruiz Huete C, Eimil M, Borrue C, Del Val J, Lopez-Manzanares L, Rojo Sebastian A, Marasescu R (2016). The high prevalence of impulse control behaviors in patients with early-onset Parkinson’s disease: a cross-sectional multicenter study. J Neurol Sci.

[CR102] Vitale C, Santangelo G, Trojano L, Verde F, Rocco M, Grossi D, Barone P (2011). Comparative neuropsychological profile of pathological gambling, hypersexuality, and compulsive eating in Parkinson’s disease. Move Disord.

[CR103] Volkow ND, Wang GJ, Fowler JS, Thanos PP, Logan J, Gatley SJ, Gifford A, Ding YS, Wong C, Pappas N (2002). Brain DA D2 receptors predict reinforcing effects of stimulants in humans: replication study. Synapse.

[CR104] Volkow ND, Wang GJ, Telang F, Fowler JS, Thanos PK, Logan J, Alexoff D, Ding YS, Wong C, Ma Y, Pradhan K (2008). Low dopamine striatal D2 receptors are associated with prefrontal metabolism in obese subjects: possible contributing factors. NeuroImage.

[CR105] Voon V, Gao J, Brezing C, Symmonds M, Ekanayake V, Fernandez H, Dolan RJ, Hallett M (2011). Dopamine agonists and risk: impulse control disorders in Parkinson’s disease. Brain J Neurol.

[CR106] Voon V, Mehta AR, Hallett M (2011). Impulse control disorders in Parkinson’s disease: recent advances. Curr Opin Neurol.

[CR107] Voon V, Rizos A, Chakravartty R, Mulholland N, Robinson S, Howell NA, Harrison N, Vivian G, Ray Chaudhuri K (2014). Impulse control disorders in Parkinson’s disease: decreased striatal dopamine transporter levels. J Neurol Neurosurg Psychiatry.

[CR108] Voon V, Napier TC, Frank MJ, Sgambato-Faure V, Grace AA, Rodriguez-Oroz M, Obeso J, Bezard E, Fernagut PO (2017). Impulse control disorders and levodopa-induced dyskinesias in Parkinson’s disease: an update. Lancet Neurol.

[CR109] Vriend C, Nordbeck AH, Booij J, van der Werf YD, Pattij T, Voorn P, Raijmakers P, Foncke EM, van de Giessen E, Berendse HW, van den Heuvel OA (2014). Reduced dopamine transporter binding predates impulse control disorders in Parkinson’s disease. Move Disord.

[CR110] Vriend C, Pattij T, van der Werf YD, Voorn P, Booij J, Rutten S, Berendse HW, van den Heuvel OA (2014). Depression and impulse control disorders in Parkinson’s disease: two sides of the same coin?. Neurosci Biobehav Rev.

[CR111] Vriend C, Raijmakers P, Veltman DJ, van Dijk KD, van der Werf YD, Foncke EM, Smit JH, Berendse HW, van den Heuvel OA (2014). Depressive symptoms in Parkinson’s disease are related to reduced [123I]FP-CIT binding in the caudate nucleus. J Neurol Neurosurg Psychiatry.

[CR112] Vriend C, Gerrits NJ, Berendse HW, Veltman DJ, van den Heuvel OA, van der Werf YD (2015). Failure of stop and go in de novo Parkinson’s disease--a functional magnetic resonance imaging study. Neurobiol Aging.

[CR113] Weintraub D, Koester J, Potenza MN, Siderowf AD, Stacy M, Voon V, Whetteckey J, Wunderlich GR, Lang AE (2010). Impulse control disorders in Parkinson disease: a cross-sectional study of 3090 patients. Arch Neurol.

[CR114] Weintraub D, Papay K, Siderowf A, Parkinson’s Progression Markers I (2013). Screening for impulse control symptoms in patients with de novo Parkinson disease: a case-control study. Neurology.

[CR115] Weintraub D, David AS, Evans AH, Grant JE, Stacy M (2015). Clinical spectrum of impulse control disorders in Parkinson’s disease. Move Disord.

[CR116] Winstanley CA, Dalley JW, Theobald DE, Robbins TW (2004). Fractionating impulsivity: contrasting effects of central 5-HT depletion on different measures of impulsive behavior. Neuropsychopharmacology.

[CR117] Wu K, Politis M, O’Sullivan SS, Lawrence AD, Warsi S, Lees A, Piccini P (2014). Problematic internet use in Parkinson’s disease. Parkinsonism Relat Disord.

[CR118] Wu K, Politis M, O’Sullivan SS, Lawrence AD, Warsi S, Bose S, Lees AJ, Piccini P (2015). Single versus multiple impulse control disorders in Parkinson’s disease: an (1)(1)C-raclopride positron emission tomography study of reward cue-evoked striatal dopamine release. J Neurol.

[CR119] Ye Z, Altena E, Nombela C, Housden CR, Maxwell H, Rittman T, Huddleston C, Rae CL, Regenthal R, Sahakian BJ, Barker RA, Robbins TW, Rowe JB (2014). Selective serotonin reuptake inhibition modulates response inhibition in Parkinson’s disease. Brain J Neurol.

[CR120] Ye Z, Altena E, Nombela C, Housden CR, Maxwell H, Rittman T, Huddleston C, Rae CL, Regenthal R, Sahakian BJ, Barker RA, Robbins TW, Rowe JB (2015). Improving response inhibition in Parkinson’s disease with atomoxetine. Biol Psychiatry.

[CR121] Zainal Abidin S, Tan EL, Chan SC, Jaafar A, Lee AX, Abd Hamid MH, Abdul Murad NA, Pakarul Razy NF, Azmin S, Ahmad Annuar A, Lim SY, Cheah PS, Ling KH, Mohamed Ibrahim N (2015). DRD and GRIN2B polymorphisms and their association with the development of impulse control behaviour among Malaysian Parkinson’s disease patients. BMC Neurol.

